# The Delivery of Stop Smoking Support to People with Mental Health Conditions: A Survey of NHS Stop Smoking Services

**DOI:** 10.1186/1472-6963-10-179

**Published:** 2010-06-24

**Authors:** Lisa McNally, Elena Ratschen

**Affiliations:** 1 Public Health Department, Surrey NHS Primary Care Trust, Cedar Court, Leatherhead, Surrey, KT22 9AE, UK; 2 UK Centre for Tobacco Control Studies, University of Nottingham, Clinical Sciences Building, City Hospital, NG5 1PB, Nottingham

## Abstract

**Background:**

People with mental health problems exhibit smoking rates up to three times that of the general population. Metabolic interactions between hydrocarbon agents in tobacco smoke and some antipsychotic drugs account for a change in medication metabolism on stopping smoking, and potentially for increased blood levels. Nicotine withdrawal can mimic or exacerbate symptoms of mental illness. Therefore, appropriate screening for mental health problems and liaison with local mental health care providers should be a priority for NHS Stop Smoking Services. The present study aimed to examine this issue through surveys with NHS Stop Smoking Service staff in London.

**Methods:**

Semi-structured telephone interviews were conducted with one senior staff member from 27 of the 29 NHS Stop Smoking Services in London.

**Results:**

It was found that only a minority of services routinely check the mental health status or mental health service use of their clients. In addition, most services do not routinely implement special checks or actions when mental health problems are revealed. It was notable that respondents reported a lack of strategic drivers supporting work with mental health patients (such as targets relating to successful quits) as well as a low level of partnership working with local mental health care providers.

**Conclusions:**

NHS Stop Smoking Services may not be operating appropriate procedures for supporting people with mental health problems. There is a need for local protocols to be implemented that include routine screening for mental health issues and liaison with mental health care providers.

## Background

Patients with mental illness are up to three times more likely to be smokers than the general population, with smoking prevalence reaching figures of 70% and above for some severe disorders, such as schizophrenia [1]. Smokers with mental illness have also commonly been found to display patterns of heavy smoking and severe nicotine dependence [2]. Frequent and heavy smoking has detrimental impacts on the lives of people with psychiatric conditions, particularly in terms of physical health. There is, for example, evidence that most of the considerable premature excess mortality found in patients with schizophrenia is due to cigarette smoking [3].

Despite evidence that people with mental health conditions are often as motivated to quit smoking as the general population [4], it may be that professional stop smoking support is not easily accessible for this group. For example, a large survey of NHS staff revealed that mental healthcare professionals are significantly less positive about taking a role in supporting service users to quit smoking than their general healthcare colleagues [5]. Furthermore, it has been shown that mental health professionals' knowledge in relation to smoking and its specific links with mental illness is often deficient, which could result in misconceptions and suboptimal treatment of tobacco dependence in mental health settings [6].

In the British Department of Health "NHS Stop Smoking Services Service and Monitoring Guidance 2010/11", people with mental health problems are identified as a 'Priority Population Group' [7]. In relation to mental health, the guidance recommends "Client medical history and current medication should always be asked and recorded during the first stop smoking appointment." In addition, attention is drawn to the need for National Health Service (NHS) Stop Smoking Services to collaborate with specialist mental health services and the importance of managing potential changes in the metabolism of certain psychotropic medications on smoking cessation [8].

There is very little information, however, on whether NHS Stop Smoking Services are following this guidance and on the extent to which the services provided are appropriately accommodating the needs of people with mental health problems. The present work aimed to examine this issue through surveys with NHS Stop Smoking Service staff in London. As the work aimed to evaluate the adherence of Stop Smoking Services to particular aspects of guidance cited above and procedures related to this, no ethical approval was sought for the project.

## Methods

The study utilised a cross-sectional survey design, aiming to include all London NHS Stop Smoking Services. Semi-structured telephone interviews were conducted by the lead researcher with the respective service's manager or a senior member of staff nominated by the manager as the 'lead' for mental health related work. Participants were recruited through an initial email and follow-up call. Interviews were scheduled in advance so as to allow respondents to ensure they were free to participate without interruption. The interview questions were sent to respondents by email one or two days before the interview took place. All participants were assured that their responses would be anonymised and treated in confidence.

The interview guide was drafted on the basis of consultation with experts in the field of Public Health and comprised 25 questions divided over three categories. The categories referred to the services' specific provisions to support people with mental health problems; to the role of local mental health Trusts in the provision of services; and to respondents' perceptions regarding options to improve their service to people with mental health problems in the future. In all cases, 'mental health problems' were broadly defined so as to include a range of conditions such as mood disorders, anxiety disorders and psychotic conditions. Participants were asked to respond to mainly closed questions and provide further details to their answer if appropriate. Detailed responses were recorded by taking extensive notes, which were fed back to the respondent for confirmation.

The data were anonymised, coded and analysed using SPSS version 17. Basic descriptive statistics were employed to retrieve frequencies and proportions for different response categories of the questionnaire. Detailed free text notes were transcribed into MS Word. These notes were analysed with reference to a qualitative Framework Approach [9]. The starting point for the analysis was a range of a priori issues derived from the objectives of the study (e.g. the issues of drivers, staff capacity). These, in turn, had emerged from reviews of the literature and through consultation with experts in the field. Other issues that emerged as recurrent in the data (e.g. - the role of senior management) were added to form an overall thematic index, which was then applied to an analysis of the notes. All statements were annotated with numerical codes from the index, with many statements being judged to apply to more than one index category. The most frequently recurring themes were identified and summaries of participants' views on themes derived (discussed below).

## Results

Of the 29 London services approached, 27 agreed to take part in the study. Two services were not available for participation because of significant staff restructure at the time of the survey, or because the staff member responsible for mental health related work was unavailable during the study period.

### Mental Health Status Recording

Of the 27 respondents, 9 (33%) reported that mental health status (either current or lifetime history) was routinely checked with every service user. The other services reported that mental health status was not asked about at all, or only on certain occasions or by certain advisors. Even if collected, data on mental health status was rarely recorded in a database. Only 3 respondents (11%) said they would currently be able to give figures on the proportion of their service clients with reported mental health problems.

### Targets, Funding, and Capacity

The survey responses addressed the issue of service targets (e.g. - specific and predefined numbers of successful quits or other outcomes that would indicate acceptable service performance). The survey found that only 3 of the 27 services (11%) were working towards targets relating to the treatment of people with mental health problems, with one other service just about to set such targets. These four services were also the only services (15%) reporting that specific funding had been set aside for the provision of stop smoking support to mental health patients.

Despite only a small minority of services reporting the existence of mental health specific targets or funding, 14 services (52%) did report that they had specific team members nominated as a lead for mental health work. In most cases, however, these staff members had other responsibilities, and the average estimated proportion of time spent on mental health work by these staff was 23%. In relation to specialist training, 15 respondents (56%) reported that their service team did contain at least one person who had attended a training course specifically on smoking and mental health.

### Service Delivery

Special checks or actions were reported to be routinely taken in cases of clients revealing the existence of a mental health problem by 15 services (59%). Of these, 10 services (37% of total sample) reported to routinely inform a health professional (mental health professional or GP) involved in the client's care of the quit attempt. The only other action or check taken related to psychotropic medication, with 10 services (37%) reporting to routinely ask clients about this issue.

### Engagement of Mental Healthcare Providers

With regard to services' perceptions of the engagement of their local mental health trusts in helping patients stop smoking, participants were asked how frequently their stop smoking service received referrals from the local mental health trust. In relation to community mental health teams, 18 respondents (67%) reported that referrals from community mental health teams were never or very rarely received. The same numbers were reported in relation to referrals from psychiatric in-patient units.

In relation to other factors that might indicate engagement between stop smoking services and mental health trusts, only 4 respondents (15%) reported that formal joint working in the context of a committee or a working group on stop smoking support was taking place. Overall, around three quarters of the participants subjectively rated the commitment of their local mental health trust to smoking cessation at the two lowest of four levels ("none" or "not enough"). A further quarter of the participants rated the commitment at the third level ("adequate") with no ratings at the highest level ("excellent") given.

### Improving Stop Smoking Support for People with Mental Health Problems

A final set of open-ended questions gauged participants' views on what should be changed or improved in order to improve the access and provision of stop smoking support to people with mental health problems. Analysis of the responses revealed three commonly raised issues in relation to changes and improvements. These were funding, systems of performance management and mental health trust engagement.

Concerning funding, a total of 14 participants felt that funds should be 'ring-fenced' (i.e. - dedicated and protected) for the delivery of stop smoking services to people with mental health problems. Participants expressed the view that this funding should, at least in part, be used to establish dedicated stop smoking specialists in this area, with protected time for service development and delivery.

In relation to performance management, participants raised the concerns over the effect of current, national 'quit targets' (i.e. - relating to the overall numbers of quitters reported by services). These targets, which focus on the recording of high numbers of quitters regardless of who those are, were seen as driving resources away from 'hard to reach' groups. A total of 15 participants raised the need to develop targets that are specific to people with mental health problems. It was felt that such targets would divert funds, senior support and service staff time directly to this group.

Finally, in relation to mental health trust engagement, participants highlighted that both senior level strategic input and communication with stop smoking services were lacking. A number of participants gave examples of how committed individuals among mental health trust staff were isolated and expressed the view that smoking cessation work needs better facilitation and strategic support by managers and senior 'champions' (11 participants). More joint working, communication and systematic reporting on smoking cessation activities and outcomes were also called for from mental health trusts (11 participants).

## Discussion

The findings of this survey serve to highlight some important gaps in the provision of NHS Stop Smoking Services to people with mental health problems. Firstly, the survey revealed that smoking related outcomes in relation to this sub-group are neither widely recorded nor systematically assessed. Maybe causal to this lack of data collation is a corresponding lack of incentive in the form of mental health specific targets or funding, which is likely to translate into other deficiencies, such as the lack of stop smoking specialist staff able to dedicate themselves to mental health work. Such work is urgently needed to make sure that one of the most vulnerable sections of the population is not left out in the area of tobacco dependence treatment.

As the findings from this study were drawn from London Stop Smoking Services exclusively, the results may not be generalisable to all NHS Stop Smoking Services in England. However, in view of the relatively high degree of standardisation of these services across the country and the existence of national guidelines, problems identified in this study are likely to be of relevance to services in other regions.

The self-reported failure of many services to routinely assess mental health status, or to put in place special checks or actions when mental health problems are revealed, highlight more than a lack of strategic drivers (such as targets or funding). They also point to potentially serious gaps in clinical governance. If care coordinators or other specialists involved in a client's mental health care are not routinely informed of a quit attempt, then the lacking coordination of treatments could be potentially detrimental to the patient's well being. Prescribers and other clinicians need to be aware of a client's smoking status and quit attempts to be in the position to manage the situation appropriately, by monitoring blood levels of antipsychotic medication (e.g. clozapine) following cessation, and by interpreting and treating symptoms of nicotine withdrawal which can strongly resemble symptoms of mental illness, such as dysphoria, depression, insomnia, and anxiety (8) adequately.

**Figure 1:**
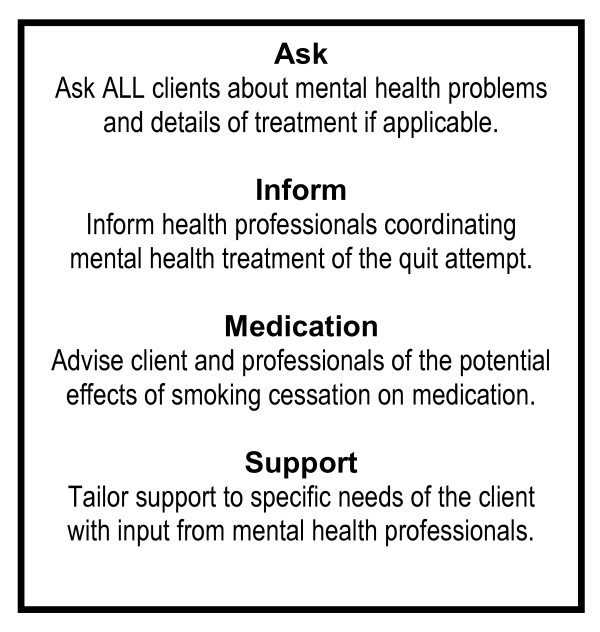
**AIMS procedure for supporting people with mental health problems to stop smoking**.

NHS Stop Smoking Services may need to consider implementing a simple procedure suitable to reduce the possibility of adverse reactions to smoking cessation among mental health service users. Such a procedure may follow an 'AIMS' structure [10] as outlined in figure 1.

The issue of poor collaboration between stop smoking and mental health services was a common theme raised in this survey. The number of respondents rating the frequency of referrals by mental health staff to be 'never or very rarely' is particularly notable. This finding suggests that either mental health trusts' engagement in the area of smoking is either poor or that it is occurring without reference to the local stop smoking service. Better collaboration between mental health and stop smoking services is likely to not only improve access to stop smoking support for mental health service users, but also facilitate the tailoring of such support to service users' needs, which, evidence suggests, leads to more effective outcomes [11].

Future research is required to examine ways in which appropriate mental health screening and liaison with mental health service providers can be best incorporated into the routine operation of NHS Stop Smoking Services. Also of value would be an investigation into the proportion of people approaching NHS Stop Smoking Services that have (or have had) mental health problems.

## Conclusions

The findings of this survey highlight a health inequality in service access. Specifically, they suggest a lack of appropriate routine procedures within Stop Smoking Services in relation to the treatment of clients with mental health problems. The findings also indicate a widespread and potentially serious lack of collaboration between key providers. There is an urgent need for guidance and better collaboration between mental health care organisations and stop smoking service providers to ensure that people with mental health problems do not remain disadvantaged in accessing and receiving adequate support in treating their tobacco dependence.

## Competing interests

LM has previously received funding to present her work from the pharmaceutical company Pfizer.

## Authors' contributions

LM designed the study and collected the interview data. ER contributed to the analysis and interpretation of data, as well as drafting and revising the manuscript. Both authors have given final approval of the version published.

## Pre-publication history

The pre-publication history for this paper can be accessed here:

http://www.biomedcentral.com/1472-6963/10/179/prepub
